# Addressing the Gender Gap in Academic Success: A History of the University of North Carolina Association of Professional Women in the Medical Sciences

**DOI:** 10.7759/cureus.49660

**Published:** 2023-11-29

**Authors:** Christina Shenvi, Amelia Drake, Etta Pisano, Susan Girdler, Wanda Nicholson, Kim Boggess

**Affiliations:** 1 Emergency Medicine, UNC (University of North Carolina) at Chapel Hill, Chapel Hill, USA; 2 Otolaryngology, UNC (University of North Carolina) at Chapel Hill, Chapel Hill, USA; 3 Radiology, American College of Radiology, Reston, USA; 4 Psychiatry, UNC (University of North Carolina) at Chapel Hill, Chapel Hill, USA; 5 Prevention and Community Health, Milken Institute School of Public Health, George Washington University, Washington, DC, USA; 6 Obstetrics and Gynecology, UNC (University of North Carolina) at Chapel Hill, Chapel Hill, USA

**Keywords:** leadership and policy management, faculty development in medical education, women in medicine, gender equity, faculty development programs

## Abstract

Despite efforts at many institutions, nationally, women still lag behind their male counterparts in leadership, promotion, and seniority. In this paper, we describe the efforts to improve the environment for women faculty at one large academic medical center through the creation of an Association of Professional Women in Medical Sciences. Over the years, the group has helped influence policies that directly affect women faculty, provided high-yield programming on topics related to women’s success, and created interprofessional networking opportunities for women faculty. We describe the challenges and successes of this group to serve as a model and inspiration for other institutions.

## Introduction

The landscape of the medical workforce is changing. In 1980, women comprised 25% of medical student graduates. In 2000, this number was 43%. The distribution of academic faculty rank by gender also changed during this time, but the proportion of women in academic medicine has persistently lagged behind that of men [1]. In 2015, despite comprising 46% of medical student graduates, women made up only 39% of full-time academic faculty, with only 22% of those at the rank of professor, and 15% serving as department chairs [1]. The reduction in women in higher-ranking positions is often referred to as the “leaky pipeline.”

Despite decades of attention and research, gender inequity in medical academia persists. A 2021 meta-analysis and systematic review of over 200 research articles found that compared to men, women were approximately three times less likely to achieve the rank of full professor compared to their male counterparts. This gender gap persisted even after adjusting for time in practice, specialty, number of publications, h-index, holding a PhD, and institution (adjusted OR: 0.55, 95% CI: 0.96-0.320) [2]. Reasons for gender inequity in academic medicine are complex and multifactorial and include the persistence of traditional gender roles, sexism in the academic environment, lack of role models, and low rates of effective mentorship, among other factors [3,4]. In 2020, Richter and colleagues studied a sample of over 550,000 medical school graduates over a 35-year period and found that women were less likely to be promoted or to be named department chair and that this inequity did not diminish over time [5]. In this paper, we describe the pathway and strategic successes that have promoted more equitable workplace policies at a large, public, academic institution through the creation of an academic women’s professional group. The group evolved from providing support at the individual level, to advocating for institutional policy changes, to becoming an established association providing ongoing faculty development. This model may serve as a template or model for other institutions and as a chronicle of the evolution of the challenges and successes that women faculty in medical sciences have experienced in the last four decades.

## Materials and methods

Here, we describe the creation of the “Women in Medicine” group and its early challenges and opportunities. In 1980, as the number of women joining the medical workforce was increasing, several junior women faculty at the University of North Carolina (UNC) at Chapel Hill School of Medicine approached the few senior women faculty for guidance on how to successfully advance their careers, and the UNC “Women in Medicine” group was born. Although this effort represented extra time, after already long clinical hours, the senior women faculty knew the academic medicine landscape was changing. They astutely recognized the need to provide critical information to their junior women colleagues who had joined the academic medical workforce. The evolution of this group and the resultant career support provided to academic women faculty demonstrates a successful response to the changing landscape of academic medicine to create a culture of mentorship and provide benefits to several generations of women faculty.

The initial effort of the “Women in Medicine” group was small. Meetings were interactive and provided a forum for women faculty to discuss the challenges of negotiation, childcare, breastfeeding, and salary issues in a safe environment with like-minded peers. The early meetings did not have speakers. The women faculty had questions to ask of each other, and the discussions were animated, supportive, and productive.  Peer mentorship, which does not adhere to the traditional hierarchies, is thought to be more accessible for underrepresented groups, including women [6,7]. During the early years, the Women in Medicine leadership was mostly self-appointed. The group increased in size from 25 to more than 60. The group began to invite formal speakers who included not just the women members but other School of Medicine leaders and deans.

Dr. Fischer, a founding senior member, recognized that mentorship is a critical element of the academic advancement of women and promotes professional development. Having a mentor or mentors improves career satisfaction, increases academic productivity, and increases chances for promotion [8]. Part of her motivation to create the Women in Medicine group was to address the limited mentorship for female faculty [3]. In 2009, the Association of American Medical Colleges (AAMC) also recognized the importance of enabling women to thrive in academics and formed the Group on Women in Medicine and Science (GWIMS) as an official group.

## Results

**Figure 1:**
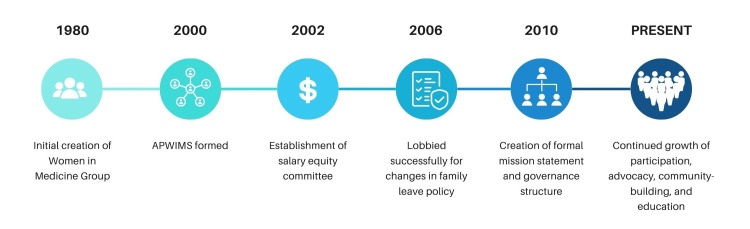
Abbreviated timeline of major events and milestones in the evolution of the UNC Association for Professional Women in Medical Sciences UNC: University of North Carolina, APWIMS: Association of Professional Women in the Medical Sciences.

Over the years, the Women in Medicine group evolved and won strategic institutional and policy successes. While the early Women in Medicine group helped provide support for individual faculty through mentorship, by the early 2000s, group leaders began to advocate formally for institutional policy changes to promote the retention and success of women faculty. The group also expanded to include women across health sciences at UNC, including Public Health, Nursing, Dentistry, and Pharmacy, and hence the name was changed to the Association of Professional Women in the Medical Sciences (APWIMS). An abbreviated timeline of key milestones is shown in Figure 1.

In 2002, another APWIMS accomplishment was the establishment of the UNC School of Medicine Salary Equity Committee. From 2002 to 2010, Dr. Pisano chaired the committee which reviewed all School of Medicine faculty salaries and made recommendations to department chairs for equity adjustments. Controlling for rank and tenure status, women faculty consistently earned less than men. As a result of this committee, roughly 10-30 faculty received a salary increase. Also, during that time, the UNC School of Medicine Dean’s Office began to provide financial support for APWIMS.

In 2006, with the support of the UNC School of Medicine Vice Dean for Academic Affairs, APWIMS successfully lobbied the University Provost to change both the university family leave policy and adopt the practice of stopping the tenure clock for faculty with major life changes (birth or adoption of a child, eldercare responsibilities, etc) to allow faculty to have more time to achieve tenure. These policies and practices remain in effect today. Dr. Etta Pisano, an APWIMS leader, also chaired the University's Committee on the Status of Women and helped APWIMS achieve institutional changes by meeting regularly with the Chancellor and Provost to lobby for the consideration of the impact of life events on all faculty, but particularly women. Her meetings led to the creation of a campus-wide parental leave policy. The University Provost at the time was receptive to the request to amend the leave policy, despite push-back from department chairs.

In 2010, Amelia F. Drake, as Executive Associate Dean of Academic Programs, was appointed to oversee APWIMS. Under her leadership, APWIMS created a mission statement and formalized its leadership structure with a president and vice president. She advocated for faculty development stipends for APWIMS leadership to attend development conferences related to the promotion of women faculty. During this time, an alumnus of the School of Medicine, Dr. Lianne DeSerres, donated financial resources to support five years of speakers and additional robust programming (a sample of which is shown in Table 1) that would address key issues women face in medical sciences.

**Table 1:** A sample of seminars, workshop, or panel topics of recent APWIMS events These occurred under the leadership of APWIMS presidents Drs Drake, Patterson, Girdler, Nicholson, and Shenvi. APWIMS: Association of Professional Women in the Medical Sciences.

Theme	Sessions Held
Workplace issues related to women in medical sciences	Workshop: Sexual harassment training; Seminar: Achieving health equity through research and clinical care; Seminar: Advancing the ethical workplace
Career advancement for women	Webinar: Negotiation skills; Seminar: Empowering your promotion; Half-day retreat: Career pathways in medicine
Career success for women in academic medical sciences	Full-day leadership development workshop for women faculty; Half-day time management retreat: Empowering your time; Dinner workshop: Navigating power dynamics and building a developmental network; Panel: A day in the life of academics; Seminar: Managing burnout; Seminar: Fatigue, moral injury, and resilience

Throughout its evolution, a common challenge that was brought up by women in APWIMS was the stress of managing a career along with the role of family caregiving. To help address this challenge, in 2016, two APWIMS leaders, Drs. Amelia Drake and Susan Girdler, successfully competed as co-Principal Investigators for, what was at the time, an experimental program developed by the Doris Duke Charitable Foundation. The purpose of the grant was to provide supplemental research support for early career Schools of Medicine (SOM) physician-scientists who had substantial family caregiving responsibilities. The UNC SOM was one of only 10 national sites to receive this five-year award, which in 2021 was renewed for another three years. In 2021, in response to the impact of the COVID-19 pandemic on women faculty, the Doris Duke Charitable Foundation also awarded Drs. Girdler and Drake a two-year grant to support early career physician-scientists to help them reclaim research time and productivity lost due to the pandemic. The most common and most helpful use of these funds by physician-scientists has been to buy out some clinical time or to hire a research assistant or program manager to maintain their research activity during the pandemic when family demands increased.

Several peer-reviewed publications have described the effect of this national collaboration with the 10 funded institutions, highlighting the impact of networking and mentoring programs and the community that the program created [9,10]. The community created through the grant and its collaborations helped validate the identities and roles of the funded women faculty as both caregivers and scientists. The funding provided support for them to enact practical solutions to help them be successful in both roles.

From 2015 onward, in addition to continuing to advocate for policies that enable women faculty to thrive and advance in their careers, APWIMS has provided regular workshops, retreats, and seminars that address topics that particularly impact women faculty (Table 1). During the COVID-19 pandemic, meetings were held virtually, under the leadership of Amelia Drake, Wanda Nicholson, and Christina Shenvi which allowed participation in much greater numbers, reaching close to 300 attendees.

## Discussion

Here, we describe one example of a successful group created to support women in academic medical sciences. While it started out as a small group of women meeting to provide interpersonal support, it has evolved to engage in important policy discussions and to provide professional development and networking opportunities for women across disciplines in medicine and medical sciences. During the last decade, APWIMS evolved to become an organization to facilitate the development of all women in the School of Medicine through information sharing, networking, and career development opportunities, and support equitable practices in the promotion of academic and health system leadership.

In the future, APWIMS will need to continue to evolve. The paucity of women at higher ranks to serve as role models, mentors, and sponsors for other women faculty needs to be addressed. Senior faculty, both male and female, need to build alliances that support women faculty at all levels, provide meaningful mentorship, and continue to advocate for policies that will enable women faculty to advance in leadership and academic rank. Medical school leaders need to prioritize gender equity among faculty and pay attention to progress being made. They should also ensure there is mentorship that addresses the needs of faculty from under-represented or marginalized backgrounds.

Nationally, there remains a need for women-focused programming and mentorship. A 2017 survey of faculty from 24 medical schools found that almost 40% of these institutions reported no special programming to promote academic success for women faculty, citing that such programming was unnecessary [11]. In 2018, a national faculty survey reported that male faculty were more likely to hold senior leadership positions (OR = 0.49; 95% CI, 0.35-0.69) [12]. These data affirm the continued need for multi-level efforts to advance the medical careers of women faculty and to support gender equity.

During the COVID-19 pandemic, the imperative for support of women in medicine and science became even more apparent. During the early pandemic, women carried more of the caregiving and family burden caused by widespread shutdowns, and their productivity in research, clinical practice, and teaching were all negatively impacted [13]. As a result, they had a greater reduction in papers submitted as first or corresponding authors [14]. Because of the increased disparities caused by the pandemic, it is even more important for institutions to support women and their career growth to ensure career productivity and longevity [15]. A group for women in medical sciences is one way to accomplish this goal.

## Conclusions

In the post-pandemic era, more than ever, it is important for institutions to support the careers of women in medical sciences. The UNC School of Medicine APWIMS’ growth and success over the past 40 years demonstrates the impact of such a group on academic faculty advancement. The group has achieved success through the development of a network of women leaders, advocacy for policies that advance women's careers, and faculty development sessions on important topics to promote women's success, among other activities. In the coming decades, APWIMS will continue to provide support for the School of Medicine women faculty, facilitate communication among women faculty and graduate and medical students, increase awareness of issues that significantly impact women, promote recruitment and retention of women at all academic ranks into leadership positions, advocate for gender equity, and promote interaction and supportive communication among women faculty and students from all health affairs on campus. We hope that more institutions will follow a similar path to promoting the success of women in academic medicine and that in the next 40 years, we will witness significant changes in the national landscape of academic medicine and medical leadership.
